# O-39 - EMERGENT VECTOR BORNE DISEASES DON’T RESPECT BOUNDARIES – A NEED FOR ONE HEALTH IN ACTION

**DOI:** 10.1093/pubmed/fdag046.035

**Published:** 2026-07-09

**Authors:** Dr Zoe Willems, Prof Dominic Mellor, Dr Kate Smith, Dr Sally Mavin, Dr Sophie El-Nahas, Sarah McLeod, Prof Jim McMenamin, Prof Catherine Moore, Dr Luis Morelo, Dr Rachel Cloke

**Affiliations:** Public Health Scotland; Public Health Scotland; Public Health Scotland; Scottish Microbiology Reference Laboratory; NHS Ayrshire and Arran; Scottish Government; Public Health Scotland; Public Health Scotland; Scottish Government; NHS Ayrshire and Arran

## Abstract

**Background:**

Emerging Vector Borne Diseases cannot be dealt with by any one institution or country alone. One Health advocates collaborative working to prevent, prepare for and respond to disease threats. ‘On paper’ this is the best approach, but how does that work in reality? Silos still exist, and not just those demarcated by geographical boundaries. Organisations have competing priorities and funding is not allocated directly to One Health. Our institutional health and funding structures are allocated to disciplinary, syndromic and departmental structures. Like many complex biological systems, vector borne diseases are just one example of emerging health threats that do not respect any of these boundaries.

**Objectives:**

To refine and enhance One Health working in Scotland.

**Methods:**

In recent years, there have been complex incidents of emergent Vector Borne Disease in Scotland: mosquito borne Usutu Virus in Blackbirds on the Isle of Arran, and human cases (probable and confirmed) of Tick Borne Encephalitis Virus complex. We summarise the incident response, risk assessment, challenges, barriers, what went well and lessons learnt.

**Results:**

These incidents required cross border, multi-agency response, coordination and communication for which there was no formal process or oversight. In spite of this, in each instance, an ad-hoc coalition of the willing responded effectively. However, we have identified recurrent gaps and inefficiencies which need to be addressed.

**Conclusions:**

In general, putting One Health into action is complex but not insurmountable. Technological innovation, directed by educated and measured human intelligence, and comprehensive interdisciplinary scientific input, offers great potential. No single organisation can lead. Recent emerging vector borne disease experience in Scotland highlights the need for dedicated resources for integrated surveillance, preparedness and response across human, animal and environmental sectors. This should be steered and managed by an oversight group formed of key One Health stakeholders, with clear strategic and operational deliverables.

